# Statins, Mortality, and Major Adverse Cardiovascular Events Among US Veterans With Chronic Kidney Disease

**DOI:** 10.1001/jamanetworkopen.2023.46373

**Published:** 2023-12-06

**Authors:** Odeya Barayev, Chelsea E. Hawley, Helen Wellman, Hanna Gerlovin, Whitney Hsu, Julie M. Paik, Ernest I. Mandel, Christine K. Liu, Luc Djoussé, J. Michael Gaziano, David R. Gagnon, Ariela R. Orkaby

**Affiliations:** 1 Ben Gurion University of the Negev, Be’er Sheva, Israel; 2 New England Geriatric Research Education and Clinical Center, Bedford and Boston, Massachusetts; 3 Massachusetts Veterans Epidemiology Research and Information Center (MAVERIC), VA Boston Healthcare System, Boston; 4 VA Boston Healthcare System, Department of Pharmacy, Boston, Massachusetts; 5 Division of Pharmacoepidemiology and Pharmacoeconomics, Department of Medicine, Brigham and Women’s Hospital, Harvard Medical School, Boston, Massachusetts; 6 Division of Renal Medicine, Department of Medicine, Brigham and Women’s Hospital and Harvard Medical School, Boston, Massachusetts; 7 Section of Geriatrics, Department of Medicine, Stanford University School of Medicine, Stanford, California; 8 Geriatric Research Education and Clinical Center, Palo Alto VA Medical Center, Palo Alto, California; 9 Division of Aging, Brigham and Women’s Hospital, Harvard Medical School, Boston, Massachusetts; 10 Department of Biostatistics, Boston University School of Public Health, Boston, Massachusetts

## Abstract

**Importance:**

There are limited data for the utility of statins for primary prevention of atherosclerotic cardiovascular disease (ASCVD) and death in adults with chronic kidney disease (CKD).

**Objective:**

To evaluate the association of statin use with all-cause mortality and major adverse cardiovascular events (MACE) among US veterans older than 65 years with CKD stages 3 to 4.

**Design, Setting, and Participants:**

This cohort study used a target trial emulation design for statin initiation among veterans with moderate CKD (stages 3 or 4) using nested trials with a propensity weighting approach. Linked Veterans Affairs (VA) Healthcare System, Medicare, and Medicaid data were used. This study considered veterans newly diagnosed with moderate CKD between 2005 and 2015 in the VA, with follow-up through December 31, 2017. Veterans were older than 65 years, within 5 years of CKD diagnosis, had no prior ASCVD or statin use, and had at least 1 clinical visit in the year prior to trial baseline. Eligibility criteria were assessed for each nested trial, and Cox proportional hazards models with bootstrapping were run. Analysis was conducted from July 2021 to October 2023.

**Exposure:**

Statin initiation vs none.

**Main Outcomes and Measures:**

Primary outcome was all-cause mortality; secondary outcome was time to first MACE (myocardial infarction, transient ischemic attack, stroke, revascularization, or mortality).

**Results:**

Included in the analysis were 14 828 veterans. Mean (SD) age at CKD diagnosis was 76.9 (8.2) years, 14 616 (99%) were men, 10 539 (72%) White, and 2568 (17%) Black. After expanding to person-trials and assessing eligibility at each baseline, there were 151 243 person-trials (14 685 individuals) of nonstatin initiators and 2924 person-trials (2924 individuals) of statin initiators included. Propensity score adjustment via overlap weighting with nonparametric bootstrapping resulted in covariate balance, with mean (SD) follow-up of 3.6 (2.7) years. The hazard ratio for all-cause mortality was 0.91 (95% CI, 0.85-0.97) comparing statin initiators to noninitiators. The hazard ratio for MACE was 0.96 (95% CI, 0.91-1.02). Results remained consistent in prespecified subgroup analyses.

**Conclusions and Relevance:**

In this target trial emulation of statin initiation in US veterans older than 65 years with CKD stages 3 to 4 and no prior ASCVD, statin initiation was significantly associated with a lower risk of all-cause mortality but not MACE. Results should be confirmed in a randomized clinical trial.

## Key Points

**Question:**

Among adults older than 65 years with chronic kidney disease (CKD) stages 3 to 4 and no history of atherosclerotic cardiovascular disease (ASCVD), is new statin use associated with lower risk of mortality or major adverse cardiovascular events (MACE)?

**Findings:**

In this cohort study of 14 828 participants, a target trial emulation of statin initiation found that statins were significantly associated with a 9% lower risk of all-cause mortality. There was a numerically lower risk of MACE, but the results were not statistically significant.

**Meaning:**

These findings suggest that in older adults with CKD stages 3 to 4 without prior ASCVD, statin initiation may lower risk of mortality and MACE.

## Teaser

This cohort study uses target trail emulation to evaluate the association of statin use with all-cause mortality and major adverse cardiovascular events among US veterans older than 65 years with chronic kidney disease stages 3 to 4.

## Introduction

Atherosclerotic cardiovascular disease (ASCVD) is the leading cause of death among older adults with chronic kidney disease (CKD).^1^ Yet, data for the role of statins for primary ASCVD prevention in individuals with moderate CKD (stages 3-4) are limited. A meta-analysis of 8834 participants in primary prevention trials with CKD, primarily stage 3, reported a 41% reduction in ASCVD events and a 34% reduction in total mortality.^2^ Conversely, there is evidence that there is no benefit to initiating statins in persons receiving hemodialysis.^3,4,5^

While some evidence supports statin use for primary prevention of ASCVD in patients with moderate CKD, few older adults were included in clinical trials,^5,6,7,8,9^ and data to guide initiation of a statin in older patients with CKD without prior history of ASCVD are needed. The lack of evidence for statins for primary prevention in older adults has contributed to a growing call to deprescribe or avoid using these medications,^6,10^ yet it is particularly the older adult population with CKD that is at the highest risk of mortality and major adverse cardiovascular events (MACEs).^11^ Therefore, we used a target trial emulation framework to examine the association of statin initiation with mortality and MACE among older adults following moderate CKD diagnosis (stages 3-4) using data from the US Veterans Health Administration.

## Methods

### Ethics

This study was approved, and the requirement for obtaining patient informed consent was waived due to its retrospective nature, by the VA Boston institutional review board. This study followed the Strengthening the Reporting of Observational Studies in Epidemiology (STROBE) reporting guideline.

### Target Trial Specification

The first step of the target trial emulation framework is to specify the hypothetical pragmatic trial protocol that would be used to address the question of interest.^12^ In our target trial we would enroll veterans with newly diagnosed moderate CKD (stages 3-4) who are older than 65 years at baseline and regular users of the Veterans Affairs (VA) Healthcare System, based on having at least 1 outpatient visit to the VA and a measurement for body mass index (BMI; calculated as weight in kilograms divided by height in meters squared) in the year prior to baseline. Individuals with any prior history of statin use before baseline would be excluded as well as those with any previous diagnosis of atherosclerotic cardiovascular diseases (ASCVD, including myocardial infarction [MI], revascularization, transient ischemic attack [TIA] or stroke, and peripheral vascular disease [PVD]), at baseline. Finally, only those without a contraindication for statin use at baseline would be included, as outlined in Table 1.

**Table 1.** Specification and Emulation of Target Trials Evaluating Statin Therapy Initiation Effectiveness on Mortality Following Diagnosis of Stage 3 or 4 CKD Using Observational Data From VA Electronic Health Records, January 1, 2005, to December 31, 2017

Protocol component	Target trial specification	Target trial emulation
Eligibility criteria	Aged >65 y with a first diagnosis of stage 3 or 4 CKD between January 1, 2005, and September 30, 2015, inclusive. No prior diagnosis of ASCVD including myocardial infarction, coronary revascularization, transient ischemic attack, stroke, peripheral vascular disease, or peripheral artery disease at baseline. No prior exposure to statins before baseline. User of VA health care system within one year prior to baseline. No enrollment in hospice. Known body mass index within 1 year of baseline. No contraindications for statin therapy: Dialysis or stage 5 CKD, Rhabdomyolysis, Active liver disease, End-stage heart failure, Active chemotherapy, radiation indicating active cancer, and End-stage dementia.	Same as for the target trial, except: Considered key criteria based on *ICD-9* and *ICD-10* diagnoses and prescriptions in the VA Corporate Data Warehouse of Centers for Medicare & Medicaid data systems. Dates for each component were set based on the earliest occurring event across the data sources.
Treatment strategies	Initiation of a new statin therapy at baseline or No statin therapy initiation at baseline.	Same as for the target trial, except: Initiation defined by receipt or fill of any statin ≥30 d supply at baseline.
Treatment assignment	Individuals randomly assigned to a treatment strategy at baseline. Individuals and their treating physicians are aware of assigned treatment.	Patients were classified according to the treatment assignment that is compatible with the observed data at baseline. Randomization was emulated by adjusting for baseline covariates.
Outcomes	Risk of death (all-cause mortality) over the follow-up period. Secondary outcome of MACE or death considered.	Same as for the target trial.
Follow-up	Follow-up starts at baseline and goes until death, outcome (specific to analysis), or the end of the study period (December 31, 2017), whichever happens first.	Same as for the target trial.
Causal contrasts	Intention-to-treat effect, ie, initiation of treatment.	Observational analogue of the intention-to-treat effect, or the prescription and fill of statin therapy within the data sources available.
Statistical analysis	Intention-to-treat analysis, Cox proportional hazards model to estimate the relative risks of outcome between treatment groups. Sensitivity analyses considered by entire analytic process to the following subgroups by: Age (66-74 and ≥75 y). Sex (male and female), Race (White, Black, and other),^a^ Cardiovascular disease risk (high and low), Frailty (frail and nonfrail), Prevalent type 2 diabetes (yes and no), Code for hyperlipidemia, and CKD stage (3 and 4).	Same as intention-to-treat analysis, except that patients may be enrolled in multiple person-trials for statistical efficiency and confidence bounds estimated using non-parametric bootstrapping. To adjust for imbalance in baseline confounders, propensity score overlap weighting was used. Sensitivity analyses conducted, in full, within the trials corresponding to each subgroup.

Abbreviations: ASCVD, atherosclerotic cardiovascular disease; CKD, chronic kidney disease; *ICD-9*, *International Classification of Diseases, Ninth Revision*; *ICD-10*, *International Statistical Classification of Diseases and Related Health Problems, Tenth Revision*; MACE, major adverse cardiovascular event; VA, Veterans Affairs.

^a^ Other race includes American Indian or Alaska Native, Asian, and Native Hawaiian or Pacific Islander individuals.

Baseline, or time zero, would be defined as the date of randomization to initiating or not initiating statins. Only person-trials within the first 5 years following CKD diagnosis would be considered, and individuals who progressed to CKD stage 5 or requiring dialysis would be excluded, as these are considered contraindications for statin initiation. Those in the treatment group, or statin initiators, would receive at least one 30-day supply of a statin at the time of randomization. Those randomized to noninitiation would not receive any statins at the time of randomization, and both the clinician and patient would be aware of treatment assignment.

The outcome for the target trial would be ascertained as the first date of death or end of study, whichever occurs first. In an analysis for secondary outcomes of MACE, the outcome would include any occurrence of MI, TIA, stroke, coronary revascularization, or death during follow-up. Time to MACE outcome would be measured as the first occurrence of a MACE, and follow-up would end at this date or end of study, whichever comes first.

In a target trial study of statin initiation, the intention-to-treat analysis would include those who filled a statin prescription at baseline, while inclusion in the per-protocol analysis would be based on the patient’s adherence to taking the medication as directed.^13^ The intention-to-treat effect size would be estimated by comparing the risks of the randomization to statin initiation vs not or treatment assignment to which the individuals were assigned at baseline. These risks would be calculated using the Cox proportional hazards model.

We additionally would conduct the following subgroup analyses by stratifying on key factors: age (66-74 vs ≥75 years), sex, race (Black or African American, White, other [American Indian or Alaska Native, Asian, or Native Hawaiian or Pacific Islander], vs unknown), specific CKD stage (3 vs 4), risk of ASCVD, frailty, and prevalent type 2 diabetes and hyperlipidemia.

### Target Trial Emulation

To emulate the previously described target trial, we used a nested trials design with data from the VA Corporate Data Warehouse (CDW)^14^ linked to Centers for Medicare & Medicaid Services (CMS) data to ensure complete capture of diagnosis data and medication use. The time frame for the study considered eligible person-trials every quarter year (3 months) from January 1, 2005, through September 30, 2015, with follow-up through the end of 2017. All target trial protocol components were the same in the emulated nested person-trials, with specific details outlined in Table 1 and later in this section.

To be considered regular VA users, individual person-trials were assessed for at least 1 clinical visit at VA in the prior year, as measured by a record in the outpatient domain or having a weight measurement in the vitals domain (for calculation of BMI). CKD stage 3 or 4 was identified by at least 1 inpatient or 2 outpatient diagnoses, defined by *International Classification of Diseases, Ninth Revision* (*ICD-9*) codes 585.3 and 585.4 or *International Statistical Classification of Diseases and Related Health Problems, Tenth Revision *(*ICD-10*) codes N18.3 and N18.4.^15,16^

Veterans with a CKD stage 3 or 4 diagnosis entered the cohort, and their index date was assigned as soon as they had been either a user in VA for a full year or turned 66 years. Patients with any statin use or diagnosis of ASCVD at any time prior to the index date were excluded. ASCVD was defined as a history of MI, TIA, stroke, PVD, or coronary revascularization using a previously published algorithm.^17^

### Exposure and Outcomes

We identified statin prescriptions using their US generic and trade names and adjudicated drug names in VA pharmacy and CMS records.^17^ The primary outcome was all-cause mortality extracted from the VA Information Resource Center Death Ascertainment File with cause-of-death and date validation from the National Death Index.^18^ In a secondary analysis of MACE, the outcome was set to the first date of a MACE event occurring after baseline for each trial using both VA data and CMS claims.

### Variables

Age, sex, and race^19^ were derived from data in the patient domain of CDW. Race categories were determined according to self-reported responses to fixed categories on enrollment in the Veterans Health Administration: American Indian or Alaska Native, Asian, Black or African American, Native Hawaiian or Pacific Islander, White, and unknown. Smoking status at baseline was derived using a validated VA algorithm.^17^ Claims codes were used to determine comorbidities associated with cardiovascular risk and/or potential considerations for or against statin prescription, including arthritis, atrial fibrillation, cancer, chronic obstructive pulmonary disease (COPD), dementia, depression, diabetes, hyperlipidemia, hypertension, and liver disease. Frailty was defined according to the 31-item VA Frailty Index.^20^ Polypharmacy was defined as having prescriptions from 5 or more VA medication classes prior to baseline.^17^ In veterans with available data, we extracted laboratory data to calculate estimated glomerular filtration rate (eGFR). Calculations were based on the serum creatinine closest to the CKD diagnosis date and did not factor race into the equation.^21^ Finally, to quantify ASCVD risk, which directly leads clinicians to recommend a statin prescription, we calculated cardiovascular risk according to the VA ASCVD risk score.^22^

### Statistical Analysis

For the hypothetical trial, we would try to randomize individuals such that all factors that could possibly affect the outcome would be balanced between the treatment groups. In the emulation setting, there is the potential for confounding by indication, thus we used propensity score weighting methods to ensure the treatment groups, conditional on these confounders, would be exchangeable at baseline. Variables used in the propensity score calculation included age, sex, race, BMI, smoking status (current vs former or never), blood pressure medications (yes vs no), nonstatin prescriptions (yes vs no), polypharmacy (yes vs no), comorbidities as listed previously (yes vs no for each), specific CKD stage (3 vs 4), Frailty Index,^20^ calendar date (as months since January 2005) to account for changes in practice, and time since first moderate CKD diagnosis (in months). Presence of a hyperlipidemia code was not included in the final propensity score model, as those with this diagnosis code should be receiving a statin per standard practice guidelines. Thus, it is not possible to disentangle reasons for a patient to have a diagnostic code without statin use, and we considered target person-trials within strata of those with and without the code instead.

We used overlap weighting to minimize the influence of extreme propensity scores on the model output.^23,24,25^ We used the observational analogue of the intention-to-treat analysis with the hazard ratios (as described in the protocol) and a nonparametric bootstrapping percentile method, with 500 bootstraps, to calculate the 95% CIs, to account for multiple person-trials per individual in the data. For each stratified analysis outlined in the target trial specification, the entire process of propensity score calculation and risk estimation, including the bootstrapping, was repeated within each subgroup.

As a final sensitivity analysis, we considered a new user design, assuming eligibility assessment at CKD diagnosis and selecting the first eligible baseline for each treatment and individual, ie, 1 row for statin initiators at time of statin initiation and 1 row for each eligible individual at index or date of initial CKD diagnosis.^26,27^ Overlap weights were, once again, calculated in the sample, and we considered statin initiation as a time-varying exposure in the outcome models.^17,25^ This design is indirectly related to the target trial emulation described in the main analysis, with eligibility for inclusion assessed at CKD diagnosis. Sensitivity analyses by age, sex, race, ASCVD risk score, diabetes, dementia, frailty, and CKD stage were conducted.

All analyses were conducted in SAS Enterprise Guide version 8.3 (SAS Institute). Two-sided *P* < .05 was considered statistically significant.

## Results

There were 16 694 veterans diagnosed with stage 3 or 4 CKD between 2005 and 2015, with no history of CVD or statin use prior to diagnosis. Mean (SD) age at CKD diagnosis was 76.9 (8.2) years, 14 616 (99%) were men, 10 539 (72%) White, and 2568 (17%) Black. This resulted in 265 464 nested person-trials (eFigure 1 in Supplement 1), with 5412 initiator person-trials and 260 052 noninitiator person-trials, in which person-trial quarter-years following statin initiation are not considered, ie, the numbers of individuals and person-trials are the same for the statin initiation group, while more than 1 trial may exist for a given individual in the noninitiator group. After excluding person-trials that would be considered ineligible at baseline due to incident CVD, progression to stage 5 CKD or dialysis, a baseline more than 5 years from original moderate CKD diagnosis, or no measurement of weight or BMI in the year prior to baseline, there were 14 828 individual veterans contributing to 154 167 nested person-trials. This resulted in 151 243 person-trials of noninitiators and 2924 person-trials of statin initiators that were later used in the analyses (eFigure 1 in Supplement 1).

Table 2 shows demographic characteristics at baseline for the eligible person-trials before propensity score weighting. Those who initiated a statin were younger, had a higher BMI, and were more likely to have diagnostic codes for diabetes, hypertension, and hyperlipidemia. Statin initiators were also more likely to have polypharmacy, take antihypertensives, be frail, and have CKD stage 4 (vs 3) at baseline. Statin initiation, or trial baseline date, also tended to occur closer to date of moderate CKD initial diagnosis, approximately 17 months following diagnosis, compared with the mean of roughly 22 months diagnosis among the noninitiator person-trials. Following propensity score overlap weighting, all baseline characteristics were balanced (eFigure 2 in Supplement 1). Simvastatin was the most often prescribed statin (1357 [46%]), followed by atorvastatin (973 [33%]), pravastatin (434 [15%]), and lovastatin (80 [3%]).

**Table 2.** Baseline Characteristics of Eligible Person-Trials in the Target Trial Emulation of Statin Initiation for the Prevention of All-Cause Mortality and Major Adverse Cardiovascular Events Among US Veterans Older Than 65 Years Free of ASCVD, Diagnosed With Moderate CKD, 2005 to 2015

Characteristic	Before propensity score weighting, No. (%)	
	Noninitiator person-trials (n = 151 243)	Statin initiator person-trials (n = 2924)
Age at index, mean (SD), y	76.9 (8.2)	74.1 7.0)
Sex		
Male	149 027 (98.5)	2887 (98.7)
Female	2216 (1.5)	37 (1.3)
Race		
American Indian or Alaska Native	851 (0.6)	26 (0.89)
Asian	1205 (0.8)	23 (0.79)
Black or African American	24 852 (16.4)	562 (19.2)
Native Hawaiian or Other Pacific Islander	1283 (0.9)	33 (1.13)
White	109 562 (72.4)	2069 (70.8)
Unknown or null	13 490 (8.9)	211 (7.2)
Smoking		
Current	21 022 (13.9)	386 (13.2)
Formerly	88 254 (58.4)	1752(59.9)
Never	41 967 (27.8)	786 (26.9)
Comorbidities		
Liver disease	15 993 (10.6)	218 (7.46)
Diabetes	38 906 (25.7)	1194 (40.8)
Hypertension	135 642 (89.7)	2710 (92.7)
Arthritis	84 106 (55.6)	1478 (50.6)
Cancer	46 720 (30.9)	781 (26.7)
Depression	30 682 (20.3)	611 (20.9)
COPD	37 507 (24.8)	643 (22.0)
Atrial fibrillation	15 722 (10.4)	279 (9.5)
Dementia	17 048 (11.3)	257 (8.8)
Hyperlipidemia	60 652 (40.1)	2005 (68.6)
BMI at index, mean (SD)	28.16 (5.6)	29.72 (5.7)
CKD stage		
3	133 912 (88.5)	2557 (87.5)
4	17 331 (11.5)	367 (12.6)
eGFR, mean (SD), mL/min/1.73 m^2^	42.70 (13.3)	42.38 (13.3)
Medication		
Polypharmacy (>5 medication classes)	6.39 (5.03)	7.95 (5.11)
Nonstatin lipid-lowering medications	7508 (5.0)	244 (8.3)
Antihypertensive medication use	107 962 (71.4)	2550 (87.2)
AHA/ACC 5-y risk category		
Low (<10%)	17 477 (11.8)	305 (10.6)
High (>10%)	130 621 (88.2)	2575 (89.4)
Frailty Index, mean (SD)^a^	0.14 (0.08)	0.15 (0.08)

Abbreviations: AHA, American Heart Association; ACC, American College of Cardiology; ASCVD, atherosclerotic cardiovascular disease; BMI, body mass index (calculated as weight in kilograms divided by height in meters squared); CKD, chronic kidney disease; COPD, chronic obstructive pulmonary disease; eGFR, estimated glomerular filtration rate.

^a^ The Frailty Index’s range is 0 to 1, with higher scores indicating worse frailty.

### Primary Outcome

**Figure 1.**
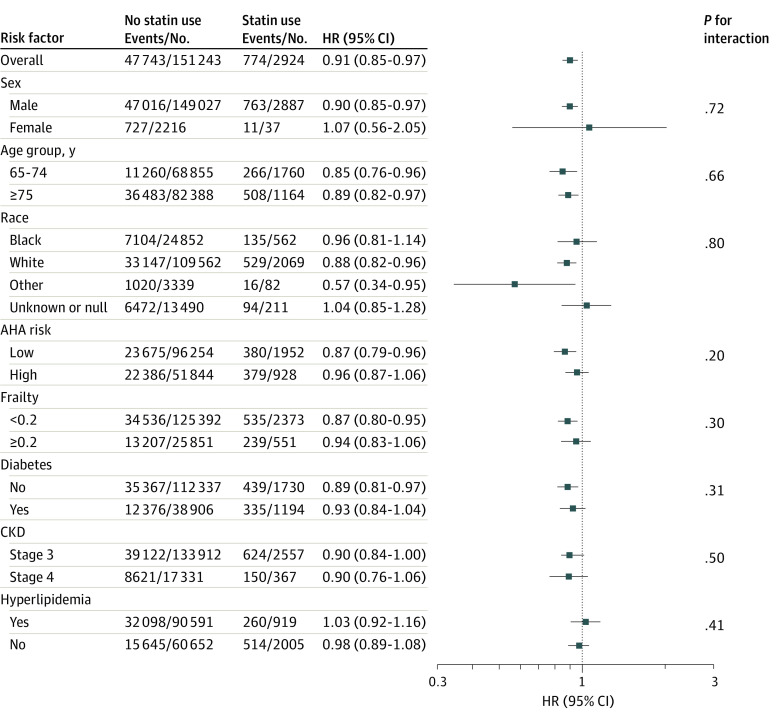
Association Between Statin Initiation and All-Cause Mortality in US Veterans Older Than 65 Years and Free of Atherosclerotic Cardiovascular Disease (ASCVD) With Stage 3 or 4 Chronic Kidney Disease (CKD) Other race includes Asian or Pacific Islander or Hawaiian or American Indian or Alaska Native. AHA indicates American Heart Association; HR, hazard ratio.

Table 3 presents the number of events among the nested person-trials and the estimated hazard ratios, with corresponding confidence intervals, for the risk of all-cause mortality and MACE outcomes in the main analyses. During a mean (SD) follow-up of 3.6 (2.7) years, there were 774 deaths among the statin initiator person-trials and 47 743 deaths in the noninitiator person-trials. The estimated hazard ratio for all-cause mortality was 0.91 (95% CI, 0.85-0.97), indicating a 9% lower risk of death for those who initiate statins within the first 5 years following moderate CKD diagnosis. Results from subgroups analyses by sex, age, race, ASCVD risk, frailty, diabetes, hyperlipidemia, and CKD stage showed a similar protective pattern for statin therapy initiators compared with noninitiators (eg, age 64-74 years: hazard ratio, 0.85; 95% CI, 0.76-0.96; age ≥75 years: hazard ratio, 0.89; 95% CI, 0.82-0.97) (Figure 1).

**Table 3.** Association of Statin Initiation and Risks of All-Cause Mortality and MACE in Eligible Person-trials of US Veterans Older Than 65 Years With Moderate CKD Diagnosis in the Previous 5 Years Between 2005 and 2017

Outcome	Events, No./person-trials at risk, No.		HR (95% CI)^a^	*P* value
	Statin initiators	Non–statin initiators		
Primary outcome: all-cause mortality	774/2924	47 743/151 243	0.91 (0.85-0.97)	.003
Secondary outcome: MACE^b^	988/2924	56 734/151 243	0.96 (0.91-1.02)	.21

Abbreviations: CKD, chronic kidney disease; HR, hazard ratio; MACE, major adverse cardiovascular event.

^a^ Confidence intervals computed using 500 nonparametric bootstrap resamples.

^b^ Time to first transient ischemic attack or stroke, myocardial infarction, revascularization, or death.

### Secondary Outcome

**Figure 2.**
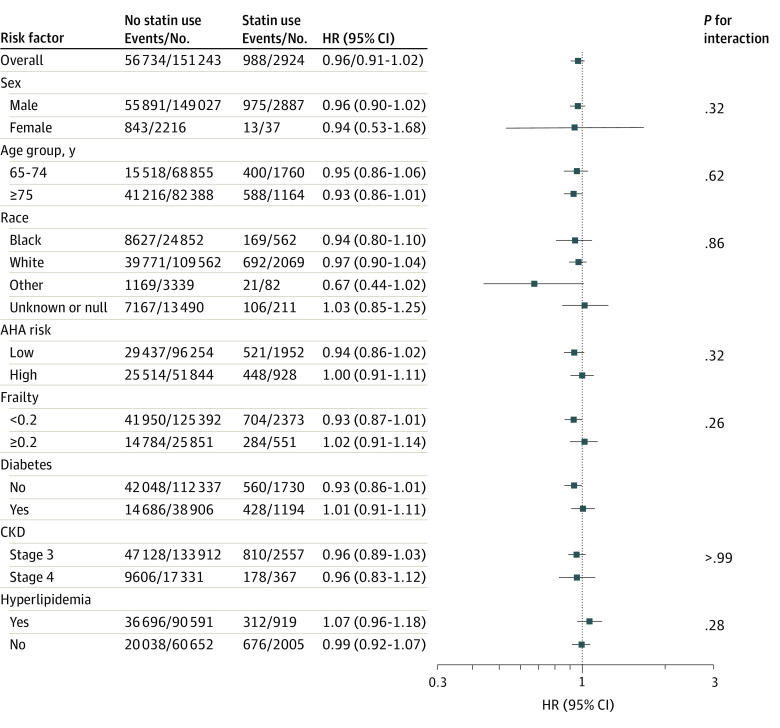
Association Between Statin Initiation and Major Adverse Cardiovascular Events in US Veterans Older Than 65 Years and Free of Atherosclerotic Cardiovascular Disease (ASCVD), With Stage 3 to 4 Chronic Kidney Disease (CKD) Other race includes Asian or Pacific Islander or Hawaiian or American Indian or Alaska Native. AHA indicates American Heart Association; HR, hazard ratio.

Over the follow-up period there were a total of 57 722 MACEs: 988 (34%) and 56 734 (38%) among statin initiator vs noninitiator person-trials. After propensity score overlap weighting, statin initiation had 4% lower risk of MACE (hazard ratio, 0.96; 95% CI, 0.91-1.02), but the results were not statistically significant (Table 3). Results remained unchanged in stratified analyses (eg, age 64-74 years: hazard ratio, 0.95; 95% CI, 0.86-1.06; age ≥75 years: hazard ratio, 0.93; 95% CI, 0.86-1.01) (Figure 2).

### Sensitivity Analysis: New User Design

When the study was expanded to include all 16 694 eligible veterans older than 65 years, free of CVD, and with a new diagnosis of CKD 3 or 4, there were 6931 deaths and 8199 MACE events. After propensity score overlap weighting was applied, the hazard ratio for mortality was 0.68 (95% CI, 0.62-0.76) and for MACE was 0.84 (95% CI, 0.76-0.92) (eTable in Supplement 1). Results were similar in stratified analyses by age, sex, race, ASCVD risk score, diabetes, dementia, frailty, and CKD stage (data not shown).

## Discussion

In this target trial emulation of statin initiation in US veterans older than 65 years with CKD stages 3 to 4 and no prior ASCVD, statin initiation was associated with a lower risk of all-cause mortality but not with MACE. In a less restrictive analysis that included all eligible veterans, results suggested a greater protective association for statins with both all-cause mortality and MACE. These results call into question the growing movement to deprescribe statins for primary prevention in older adults with CKD.^10,28^

Current Kidney Disease Improving Global Outcomes guidelines recommend initiation of statins in older patients with moderate CKD due to the established risk of ASCVD in this population.^29,30^ However, the guidelines do not differentiate between primary and secondary prevention.^31,32^

Our study supports prior literature on the role of statins for primary ASCVD prevention in CKD.^2^ A post hoc meta-analysis of 6 randomized clinical trials assessing 8834 adults with CKD stages 1 to 3 reported a 34% reduction in mortality and 41% reduction in cardiovascular events among new statin users vs nonusers. These pooled effect sizes are greater than seen in the restricted target trial emulation analysis reported here, although they are similar to the results in this report for mortality in the new user design.

Additional post hoc analyses of primary prevention trials focusing on individuals with nondialysis CKD have been mixed. One reported no statistically significant mortality benefit for statins at any stage of CKD,^33^ while 2 reported up to 51% reduction in mortality.^34,35^ However, in each of these post hoc analyses and the meta-analysis, statins were associated with a significant decreases in risk of MACE.^2,33,34,35^

Chronologic age is established as the strongest nonmodifiable risk factor for ASCVD. Coupled with the hyperinflammatory state of CKD,^36^ it is likely that older adults with CKD would benefit from statins for primary prevention. Although trial data suggest a 2- to 5-year time from initiation of a statin to benefit,^37^ current literature implies that age alone should not limit prescription.^17^ Prior work that studied 327 000 veterans aged 75 years and older free of ASCVD, irrespective of kidney disease status, reported a 25% reduction in all-cause mortality (hazard ratio, 0.75; 95% CI, 0.74-0.76) among new users of statins compared with nonusers and an 8% reduction in MACE (hazard ratio, 0.92; 95% CI, 0.91-0.94).^38^

Our study specifically assessed the role of age in addition to CKD level when considering statins for primary prevention. Results were consistent for every age group. The decision to prescribe is multifactorial, and these data support the use of statins for primary prevention for any older adult with CKD.

Clinical trials, such as the Statins in Reducing Events in the Elderly (STAREE)^39^ and Pragmatic Evaluation of Events and Benefits of Lipid-Lowering in Older Adults (PREVENTABLE)^40^ trials, will begin to address the question of statins for primary prevention specifically for older adults with CKD as well as in specific CKD stages. More research is also needed to explore the implications of our results in the prespecified subgroups that remained consistent when stratifying by age, sex, race and ethnicity, diabetes, frailty, or dialysis. However, these results were exploratory, and many subgroups were small. Existing literature has found declining benefit of statins for primary prevention for those with higher CKD stage and no evidence of benefit in persons receiving dialysis who initiate a statin for primary prevention.^5^

This study has several strengths. Our data included linked electronic health record, clinical, and claims data with more than a decade of follow up. Using this large data set, we were able to focus on the role of statins for primary prevention among older adults with CKD stages 3 to 4, a population typically underrepresented in clinical trials. An adequate event rate was ensured due to the overall greater burden of cardiovascular disease among our population of veterans compared with the general population.^41^ We used rigorous methods to control for confounding by indication, including explicit specification of the target trial and its emulation as well as restricting to regular VA users, incorporating propensity score weights, and multiple sensitivity analyses.

### Limitations

This study has several limitations. First, this is a study of US veterans who are predominantly male and White. As a result, our findings may not be generalizable to other populations. Second, there may be residual unmeasured confounding due to the nature of administrative claims data, despite the target trial emulation and propensity score methods. Third, we did not evaluate the per-protocol effect size for statin use after the initial prescription, although we have previously shown that most individuals prescribed a first statin continue to fill prescriptions for statins over time.^17^ The per-protocol design and emulation are more difficult to perform, as individuals may discontinue treatment due to adverse effects, and sometimes those receiving a prescription fill may not adhere to treatment.^42,43,44^ Fourth, we did not evaluate the dose or duration of statin therapy during their follow-up period. Fifth, simvastatin was the most common statin in this study but is less potent and is used less frequently in current practice.^17^ However, simvastatin is less potent than atorvastatin or rosuvastatin, which are more commonly prescribed. It is possible that a study in patients taking these statins might show associations with even further reduction in outcomes among statin users vs nonusers.^17,45^

## Conclusions

Among US veterans older than 65 years with CKD stages 3 to 4 and no prior ASCVD, statin initiation was associated with a lower risk of all-cause mortality compared with no statin initiation. Results should be confirmed in a randomized clinical trial. However, until such trials are completed, these data argue against withholding or deprescribing statins for primary prevention in older patients with CKD stages 3 to 4.

## Notes

Supplement 1. **eFigure 1.** Flowchart Diagram for the Target Trial Emulation of Statin Initiation and All-Cause Mortality or MACE in Veterans Older Than 65 Years Diagnosed With Moderate CKD (Stages 3 and 4) Between January 2005 and December 2015 **eFigure 2.** Covariate Balance Plot **eTable.** Association Between Statin Use vs Nonuse in 16 694 US Veterans Older Than 65 Years After Propensity Score Weighting

Supplement 2.
Data Sharing Statement
