# Supplementary material for: Statins, Mortality, and Major Adverse Cardiovascular Events Among US Veterans With Chronic Kidney Disease
Source: JAMA Netw Open. 2023 Dec 6;6(12):e2346373. doi: 10.1001/jamanetworkopen.2023.46373 (PMC10701610; doi:10.1001/jamanetworkopen.2023.46373)
Supplement: Supplement 1. — eFigure 1. Flowchart Diagram for the Target Trial Emulation of Statin Initiation and All-Cause Mortality or MACE in Veterans Older Than 65 Years Diagnosed With Moderate CKD (Stages 3 and 4) Between January 2005 and December 2015 eFigure 2. Covariate Balance Plot eTable. Association Between Statin Use vs Nonuse in 16 694 US Veterans Older Than 65 Years After Propensity Score Weighting [file jamanetwopen-e2346373-s001.pdf]

Supplemental Online Content

Barayev O, Hawley CE, Wellman H, et al. Statins, mortality, and major adverse cardiovascular events among US veterans with chronic kidney disease. *JAMA Netw Open.* 2023;6(12):e2346373. doi:10.1001/jamanetworkopen.2023.46373

eFigure 1. Flowchart Diagram for the Target Trial Emulation of Statin Initiation and All-Cause Mortality or MACE in Veterans Older Than 65 Years Diagnosed With Moderate CKD (Stages 3 and 4) Between January 2005 and December 2015

eFigure 2. Covariate Balance Plot

eTable. Association Between Statin Use vs Nonuse in 16 694 US Veterans Older Than 65 Years After Propensity Score Weighting

This supplemental material has been provided by the authors to give readers additional information about their work.

© 2023 Barayev O et al. *JAMA Network Open.*

eFigure 1. Flowchart Diagram for the Target Trial Emulation of Statin Initiation and All-Cause Mortality or MACE in Veterans Older Than 65 Years Diagnosed With Moderate CKD (Stages 3 and 4) Between January 2005 and December 2015

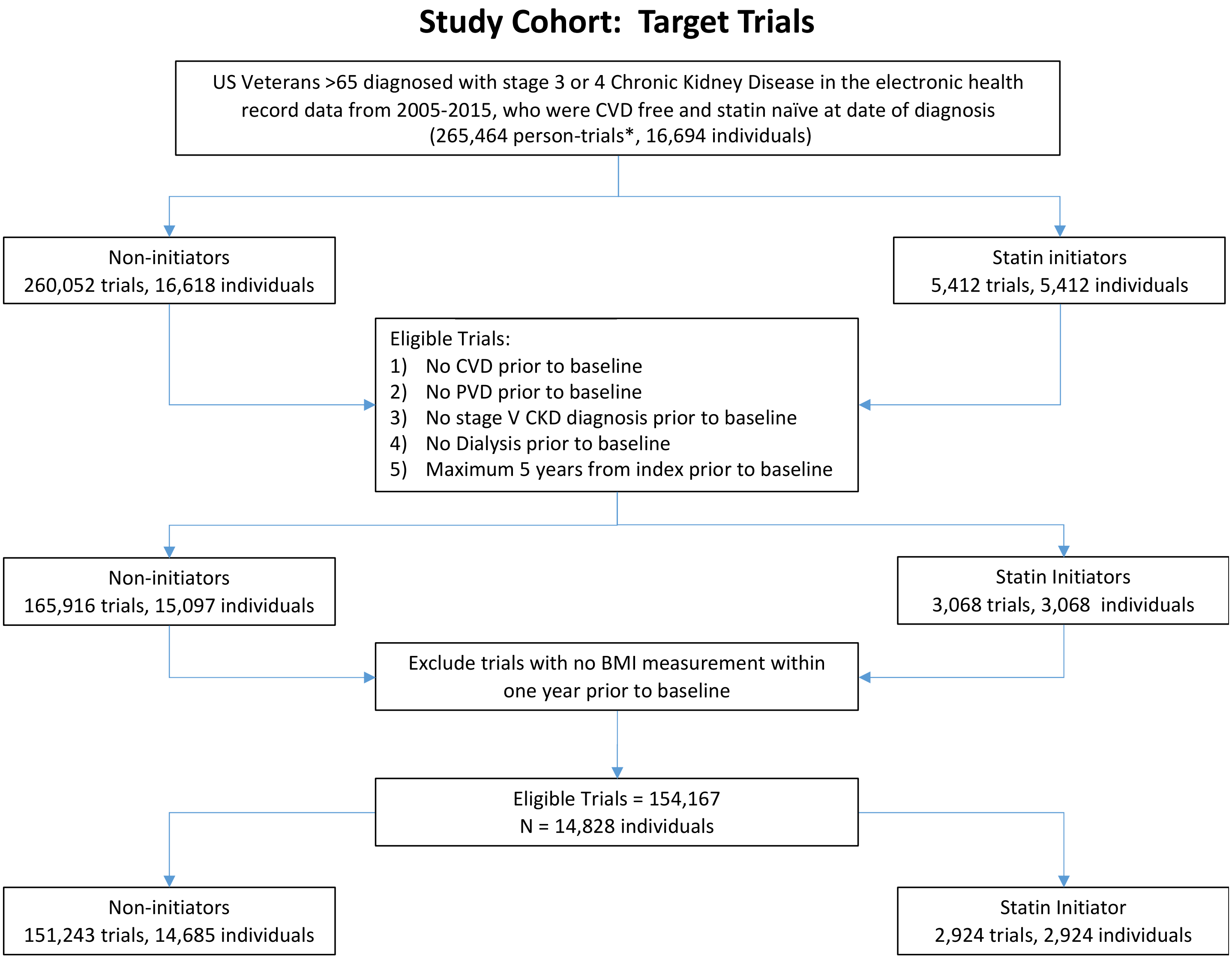

*Trials considered every quarter year per person from date of moderate CKD to first 30-day prescription of any statins.

© 2023 Barayev O et al. *JAMA Network Open.*

eFigure 2. Covariate Balance Plot

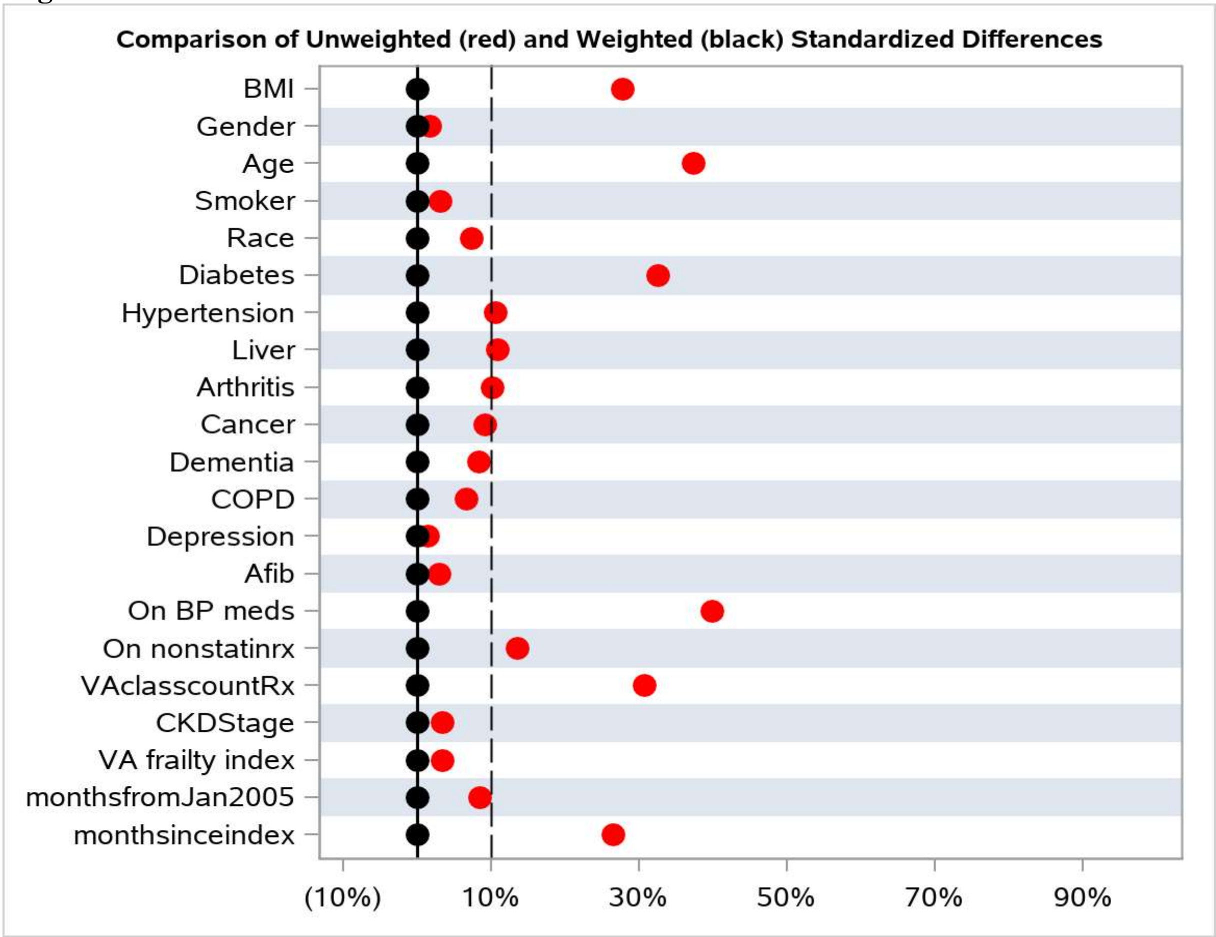

BMI = body mass index
Afib = atrial fibrillation
COPD = chronic obstructive pulmonary disease
BP = blood pressure
Nonstatinrx = non-statin medication
VAclasscountRx =
CKDStage = chronic kidney disease disease stage (3 or 4)

© 2023 Barayev O et al. *JAMA Network Open.*

eTable. Association Between Statin Use vs Nonuse in 16 694 US Veterans Older Than 65 Years After Propensity Score Weighting

	**Statin users Events/N at risk**	**Non-users Events/N at risk**	**HR (95% CI)**	**P-value**
**Primary Outcome**				
All-cause mortality	1662/5412	5269/16618	0.68 (0.62,0.76)	<0.001
**Secondary Outcome**				
Major Adverse Cardiovascular Event[a]	1678/4538	6521/16618	0.84 (0.76,0.92)	0.001

[a]Time to first TIA/stroke, MI, revascularization, or death

TIA = Transient Ischemic Attack

MI = Myocardial Infarction

© 2023 Barayev O et al. *JAMA Network Open.*
