# Supplementary material for: Statins, Mortality, and Major Adverse Cardiovascular Events Among US Veterans With Chronic Kidney Disease
Source: JAMA Netw Open. 2023 Dec 6;6(12):e2346373. doi: 10.1001/jamanetworkopen.2023.46373 (PMC10701610; doi:10.1001/jamanetworkopen.2023.46373)
Supplement: Supplement 2. — Data Sharing Statement [file jamanetwopen-e2346373-s002.pdf]

Data Sharing Statement

Barayev. Statins, Mortality, and Major Adverse Cardiovascular Events Among US Veterans With Chronic Kidney Disease. *JAMA Netw Open*. Published December 06, 2023. doi:10.1001/jamanetworkopen.2023.46373

Data
Data available: Yes
Data types: Deidentified participant data
How to access data: Data will be shared in accordance with VA Boston IRB rules.
When available: With publication

Supporting Documents
Document types: Statistical/analytic code
How to access documents: Contact: aorkaby@bwh.harvard.edu
When available: With publication

Additional Information
Who can access the data: researchers whose proposed use of the data has been approved
Types of analyses: for a specified purpose
Mechanisms of data availability: with a signed data access agreement
